# Ex Vivo Evidence for the Contribution of Hemodynamic Shear Stress Abnormalities to the Early Pathogenesis of Calcific Bicuspid Aortic Valve Disease

**DOI:** 10.1371/journal.pone.0048843

**Published:** 2012-10-31

**Authors:** Ling Sun, Santanu Chandra, Philippe Sucosky

**Affiliations:** 1 Department of Aerospace and Mechanical Engineering, University of Notre Dame, Notre Dame, Indiana, United States of America; 2 Eck Institute for Global Health, University of Notre Dame, Notre Dame, Indiana, United States of America; Brigham and Women's Hospital, Harvard Medical School, United States of America

## Abstract

The bicuspid aortic valve (BAV) is the most common congenital cardiac anomaly and is frequently associated with calcific aortic valve disease (CAVD). The most prevalent type-I morphology, which results from left-/right-coronary cusp fusion, generates different hemodynamics than a tricuspid aortic valve (TAV). While valvular calcification has been linked to genetic and atherogenic predispositions, hemodynamic abnormalities are increasingly pointed as potential pathogenic contributors. In particular, the wall shear stress (WSS) produced by blood flow on the leaflets regulates homeostasis in the TAV. In contrast, WSS alterations cause valve dysfunction and disease. While such observations support the existence of synergies between valvular hemodynamics and biology, the role played by BAV WSS in valvular calcification remains unknown. The objective of this study was to isolate the acute effects of native BAV WSS abnormalities on CAVD pathogenesis. Porcine aortic valve leaflets were subjected *ex vivo* to the native WSS experienced by TAV and type-I BAV leaflets for 48 hours. Immunostaining, immunoblotting and zymography were performed to characterize endothelial activation, pro-inflammatory paracrine signaling, extracellular matrix remodeling and markers involved in valvular interstitial cell activation and osteogenesis. While TAV and non-coronary BAV leaflet WSS essentially maintained valvular homeostasis, fused BAV leaflet WSS promoted fibrosa endothelial activation, paracrine signaling (2.4-fold and 3.7-fold increase in BMP-4 and TGF-β1, respectively, relative to fresh controls), catabolic enzyme secretion (6.3-fold, 16.8-fold, 11.7-fold, 16.7-fold and 5.5-fold increase in MMP-2, MMP-9, cathepsin L, cathepsin S and TIMP-2, respectively) and activity (1.7-fold and 2.4-fold increase in MMP-2 and MMP-9 activity, respectively), and bone matrix synthesis (5-fold increase in osteocalcin). In contrast, BAV WSS did not significantly affect α-SMA and Runx2 expressions and TIMP/MMP ratio. This study demonstrates the key role played by BAV hemodynamic abnormalities in CAVD pathogenesis and suggests the dependence of BAV vulnerability to calcification on the local degree of WSS abnormality.

## Introduction

The bicuspid aortic valve (BAV) is the most common congenital cardiac anomaly and is present in 2–3% of the general population [1]. While a normal tricuspid aortic valve (TAV) consists of three functional leaflets, a BAV is formed with only two. The most common type-I morphology results from the fusion of the right- and left-coronary leaflets and features a central fibrous raphe at the site of leaflet fusion [2], [3]. Despite its limited prevalence, the BAV malformation has emerged as the first indication for surgical valve replacement and as a major risk factor for calcific aortic valve disease (CAVD) [4]–[6]. This condition covers a spectrum of disease from initial changes in the cell biology of the valve leaflets, through early calcification, tissue remodeling and aortic sclerosis, to outflow obstruction and aortic stenosis [7]–[9]. The later stages are characterized by fibrotic thickening of the leaflets and side-specific formation of calcium nodules near the fibrosa [10].

Recent studies have evidenced the multi-faceted aspect of CAVD, which involves actively regulated processes including inflammation [8], [10], [11], osteogenesis [12]–[14], apoptosis [15], [16] and necrosis [17], [18]. Valvular inflammation, which is the hallmark of the early stage of CAVD, has been linked to the activation of the leaflet endothelium via enhanced expression of vascular cell and intercellular adhesion molecules (VCAM and ICAM, respectively) [19]–[22]. Elevated expressions of pro-inflammatory cytokines such as bone morphogenic proteins (BMPs) [23], [24] and transforming growth factor-β1 (TGF-β1) [15], [25] have also been observed in early calcific lesions, demonstrating the key role played by paracrine signaling in CAVD development. Downstream of those events, the valve interstitial cells switch from a quiescent fibroblastic phenotype to an activated myofibroblastic phenotype expressing α-smooth muscle actin (α-SMA) or to an osteoblastic phenotype marked by runt-related transcription factor 2 (Runx2) [26]–[29]. Those activated phenotypes result in the progressive loss of valvular homeostasis caused by the increased imbalance between matrix metalloproteinases and their tissue inhibitors [30], [31], and the upregulation of cathepsins [32]–[34]. The ultimate end-point of the disease, which consists of the formation of calcific lesions, is associated with an upregulation of bone matrix proteins [26], [35]–[37].

While the calcification of BAV leaflets has been historically linked to a combination of genetic predisposition and atherogenic risk factors, hemodynamic abnormalities are increasingly pointed as potential pathogenic contributors. Specifically, the expression of particular phenotypes of valvular endothelial (VECs) and interstitial cells (VICs) appears to depend not only on a combination of intrinsic genetically programmed biology [38]–[42] but also on local hemodynamic environmental factors [34], [43]–[48], one family of which is the fluid wall shear stress (WSS) resulting from the relative motion between the leaflets and the blood flow [43], [45]. *Ex vivo* studies conducted in our laboratory have evidenced the existence of shear stress-sensitive pro-inflammatory pathways and potential synergies between cytokines and cell adhesion molecules in the early valvular response to WSS alterations [46], [47]. Although those studies have demonstrated the sensitivity of leaflet tissue to WSS magnitude and pulsatility, the elucidation of the role played by native BAV WSS abnormalities in CAVD pathogenesis has been hampered by the complexity of the native BAV hemodynamic environment and the challenge to replicate it in the laboratory setting. BAV flow visualization [49], radiography [50], [51] and magnetic resonance imaging [52]–[54] have revealed the existence of an elliptical clam-shell shaped orifice, an intrinsic degree of stenosis, an eccentric systolic jet and abnormal downstream helical flow patterns. Our recently published computational work on the comparison of TAV and type-I BAV WSS [55] demonstrated that, while those hemodynamic abnormalities have little impact on the non-coronary BAV leaflet WSS, they significantly affect the pulsatility and magnitude of the WSS on the fused BAV leaflet, which is interestingly the leaflet most vulnerable to calcification [2].

In light of the existence of hemodynamic stress abnormalities on type-I BAV leaflets and the established sensitivity of valvular tissue to WSS, we hypothesized that BAV hemodynamic WSS abnormalities contribute to CAVD pathogenesis by promoting endothelial activation, pro-inflammatory paracrine signaling, VIC activation and osteogenesis. This hypothesis was tested *ex vivo* by exposing fresh porcine leaflets to the native TAV and type-I BAV WSS and by comparing their acute pathological response via immunostaining, immunoblotting and zymography. The results reveal the pathogenic potential of the native BAV hemodynamic environment and the particular vulnerability of the fused BAV leaflet to calcification.

## Materials and Methods

### Tissue Harvest and Preparation

**Figure 1:**
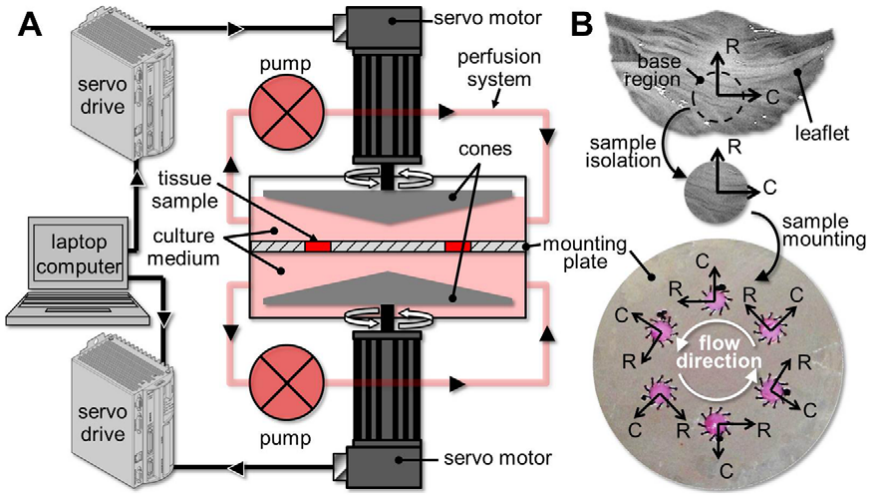
Double-sided shear stress bioreactor setup. Setup schematic (A) and tissue isolation and mounting (B) (R: radial tissue direction; C: circumferential tissue direction).

Porcine valves (6–12 months) were obtained from a local abattoir (Martin's Custom Butchering, Wakarusa, IN; permission was obtained from this slaughterhouse to use these animal parts), immediately rinsed in sterile Dubelcco's phosphate buffered saline (PBS; Sigma-Aldrich, St Louis, MO) and transported to the laboratory in ice-cold PBS. All subsequent procedures were conducted in a sterile flow hood. A circular section of 7 mm in diameter was excised from the base region of each leaflet (i.e., region most prone to calcification [10], [56]). The samples were randomized, selected from different animals and different leaflets (i.e., left-coronary, right-coronary and non-coronary) and assigned to the different treatment groups outlined below. The samples were subjected to fluid shear stress for 48 hours (duration sufficient for initial biological changes secondary to mechanical stimulation to become evident [26], [34], [45]–[47], [57]–[59]) using our previously described double-sided shear stress bioreactor [60]. This device, which consists of two cones, each rotating above one side of a tissue mounting plate, is capable of exposing simultaneously but independently both sides of six tissue specimens to differential WSS without significant stretch or flexure (Figure 1A). For each experimental run, six tissue samples were sutured to the mounting plate. The native alignment of the tissue relative to blood flow was maintained by aligning the radial direction of the samples with the direction of the cone motion (Figure 1B). The device was maintained in an incubator at 37°C and 5% CO_2_ and was continuously perfused with standard culture medium (Dulbecco's Modified Eagle's Medium supplemented with 10% fetal bovine serum, 3.7 g/L sodium bicarbonate, 0.05 g/L ascorbic acid, 10% non-essential amino acid solution and 1% penicillin-streptomycin; all from Sigma-Aldrich) at a rate of 108 mL/hour (i.e., two bioreactor volumes/hour). For each experiment, the perfusion system was totally drained and replenished with fresh medium after 24 hours. On completion of the shear stress experiments, the 7-mm circular samples were harvested, washed three times in ice-cold sterile PBS and trimmed into smaller 5-mm disks in order to isolate the central region of the tissue that was exposed to the flow and discard the peripheral region that was sutured to the mounting plate. The resulting 5-mm samples were either frozen in optimum cutting temperature (OCT) medium (Electron Microscopy Sciences, Hatfield, PA), cut into 7-µm sections using a Microm Cryo-Star HM 560MV Cryostat (Walldorf, Germany) and mounted on slides for histological and immunohistochemical analyses, or flash frozen in liquid nitrogen for immunoblotting or zymography analyses.

### Experimental Groups and Conditions

**Figure 2:**
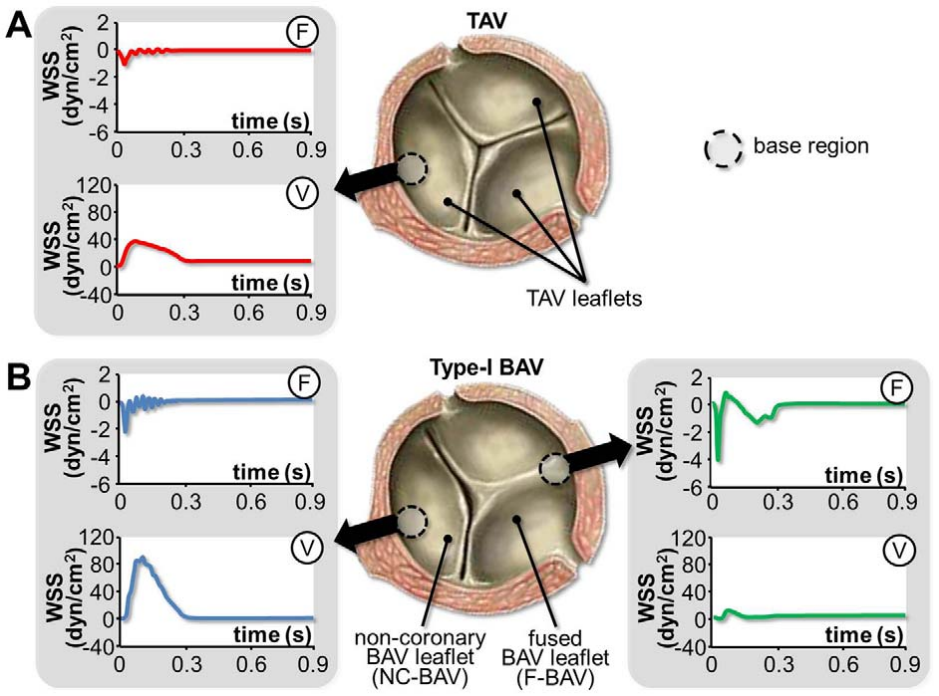
Native TAV and BAV WSS environments. Side-specific WSS waveforms predicted by fluid-structure interaction simulations in the base region of TAV (A) and type-I BAV leaflets (B) (F: fibrosa; V: ventricularis).

One control group and three mechanical treatment groups were considered in this study: 1) fresh porcine leaflet tissue (control); 2) porcine leaflet tissue exposed to TAV leaflet WSS; 3) porcine leaflet tissue exposed to fused BAV (F-BAV) leaflet WSS; and 4) porcine leaflet tissue exposed to non-coronary BAV (NC-BAV) leaflet WSS. The native WSS waveforms experienced by the fibrosa and ventricularis in the base of each leaflet (i.e., TAV, F-BAV and NC-BAV) were obtained from fluid-structure interaction simulations in TAV and type-I BAV anatomies [55]. The average WSS predicted in the base of the ventricularis of TAV, F-BAV and NC-BAV leaflets consisted of pulsatile (i.e., positive) waveforms varying between 0 and 39 dyn/cm^2^, 0 and 11 dyn/cm^2^ and 0 and 78 dyn/cm^2^, respectively, over a cardiac cycle of 0.86 s. In contrast, the average WSS predicted in the base of the fibrosa of the same leaflets consisted of oscillatory (i.e., alternatively positive and negative) waveforms ranging from −1.1 to 0.4 dyn/cm^2^, −4.5 to 1.1 dyn/cm^2^ and −0.4 to 1.8 dyn/cm^2^, respectively (Figure 2).

### Shear Stress Bioreactor Programming and Validation

**Figure 3:**
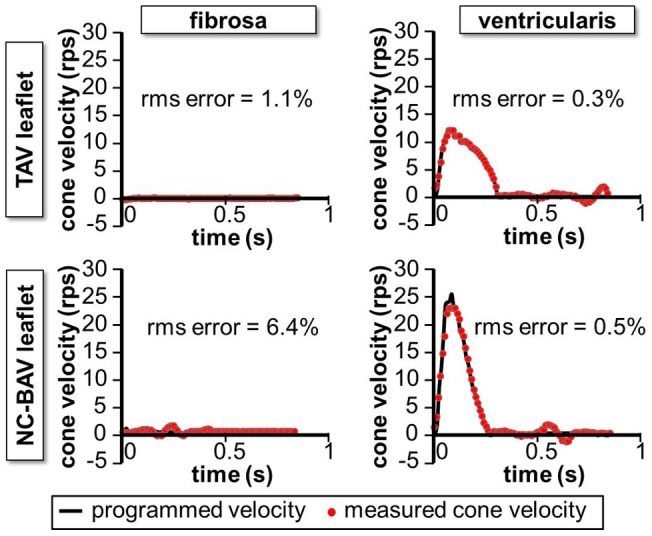
Shear stress bioreactor mechanical validation. Comparison between the programmed and measured cone angular velocities for the production of the TAV and NC-BAV ventricularis and fibrosa WSS (rms error expressed as a percentage of the maximum shear stress range experienced by the leaflet over one cardiac cycle).

The shear stress bioreactor was programmed to replicate each pair of WSS waveforms considered in this study (i.e., ventricularis and fibrosa WSS experienced by TAV, F-BAV and NC-BAV leaflets). The cone angular velocity 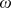 required to produce each of the six resulting WSS waveforms 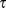 was calculated based on the previously validated WSS vs. cone velocity relationship [60]:
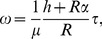
(1)where 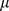 is the dynamic viscosity of the culture medium (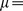
0.95×10^−3^ kg.m^−1^.s^−1^), 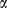 is the angle between the cone and the plate (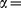
0.5°), 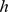 is the distance between the cone apex and the plate (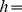
200 µm) and 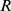 is the radius at which the tissue samples are mounted on the plate (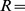
20 mm). The velocity waveforms were then programmed in their respective servo drive. While the bioreactor has been previously validated in terms of its capability to maintain desired shear stress levels and variations on both leaflet surfaces and to maintain tissue under viable and sterile conditions for up to 96 hours, its capability to accurately replicate any of the WSS waveforms considered in the present study was also investigated. For each WSS waveform, the actual angular velocity achieved by the cone was measured by recording the feedback output by the motor through a data acquisition card. The comparison between the programmed and measured cone angular velocities for the production of the WSS waveforms in the basal region of the TAV and NC-BAV leaflet is shown in Figure 3. The root-mean-square (rms) error indicated in each plot is expressed as a percentage of the maximum shear stress amplitude experienced by each leaflet. Despite a significant noise-to-signal ratio in the waveforms used to produce the fibrosa WSS, the normalized rms errors remain moderate (rms error <6.4%), which demonstrates the ability of the bioreactor to expose fresh leaflet tissue to the WSS waveforms considered in the present study.

### Biological Analyses

Hematoxylin and eosin (H&E) staining and terminal transferase dUTP nick end labeling (TUNEL) assay were performed to characterize tissue structure and cell apoptosis, respectively. Standard immunostaining, Western blot and gelatin zymography protocols were performed to detect expression/activity of the cell-adhesion molecules VCAM-1 and ICAM-1, the pro-inflammatory cytokines BMP-4 and TGF-β1, the myofibroblastic and osteoblastic differentiation markers α-SMA and Runx2, the extracellular matrix (ECM) remodeling markers cathepsin L, cathepsin S, MMP-9, MMP-2 and its tissue inhibitor TIMP-2, and the bone matrix synthesis biomarker osteocalcin. The specific focus on BMP-4 and TGF-β1 rather than on other isoforms was motivated by previous reports demonstrating the presence of those molecules in human valvular calcified lesions [23], [25] and *ex vivo* studies suggesting their particular sensitivity to valvular hemodynamic cues [44]–[47], [57], [61]. Similarly, the gelatinases MMP-2 and MMP-9 were selected based on the clinical demonstration of their increased presence and activity in calcified valves [31], [62]–[64], their established mechanosensitivity in valvular tissue [34], [45] and the suggested synergy between MMP-9 and TGF-β1 [15], [65]. Detailed descriptions of the protocols are provided below.

#### H&E Staining

Frozen tissue sections were thawed for 10 minutes at room temperature, rinsed in PBS for 10 minutes and incubated in activated hematoxylin (Electron Microscopy Sciences) for 2 minutes. Slides were washed for 5 minutes in tap water, stained with 0.2% ammonia in water for 1 minute, rinsed in tap water, counterstained for 5 minutes in eosin (Electron Microscopy Sciences), rinsed in water, then dehydrated. A resinous mounting agent was applied and the slides were coverslipped and allowed to dry overnight before viewing. H&E images were recorded using the normal white light of a Nikon E600 imaging microscope.

#### Immunostaining

Frozen tissue sections prepared as described above were first thawed to room temperature and permeabilized in acetone at −20°C. Sections were then blocked using 10% animal serum in PBS (Sigma-Aldrich), 0.2% Trixon-100 (Sigma-Aldrich) and 1% dimethyl sulfoxide (DMSO; Thermo Fisher Scientific, Waltham, MA) in 1x PBS for 1 hour at room temperature. Following the blocking step, the slides were then incubated overnight at 4°C in primary antibody in 2–10% blocker at the following dilutions: VCAM-1 (1∶50, Santa Cruz Biotechnology, Santa Cruz, CA), ICAM-1 (1∶50, Southern Biotech, Birmingham, AL), TGF-β1 (1∶25, Santa Cruz), BMP-4 (1∶150, Abcam, Cambridge, MA), cathepsin L (1∶25, Santa Cruz), cathepsin S (1∶25, Santa Cruz), MMP-2 (1∶100, EMD Millipore, Billerica, MA) MMP-9 (1∶100, EMD Millipore), TIMP-2 (1∶50, Santa Cruz) and osteocalcin (1∶150, EMD Millipore). Following primary antibody incubation, sections were washed 3 times in 1x PBS and incubated with anti-rabbit, anti-goat or anti-mouse (all from Santa Cruz) secondary antibodies at 1∶100 dilution for 2 hours at room temperature. The tissue sections were then washed 3 times in 1x PBS, counterstained with 1 4',6-Diamidino-2-phenylindole (DAPI; Sigma-Aldrich), mounted with fluorescence mounting medium (Dako, Carpinteria, CA), coverslipped and stored at 4°C. Slides were subsequently imaged under the mercury lamp of a Nikon E600 imaging microscope using a TR/FITC/DAPI filter.

#### Cell Apoptosis

Detection of cell apoptosis was performed by using a TUNEL assay kit (Roche Applied Science, Indianapolis, IN). Cryopreserved tissue sections were fixed with 4% paraformaldehyde in PBS for 20 minutes, washed with PBS for 30 minutes, permeabilized with 0.1% Triton X-100 and 0.1% sodium citrate in PBS (2 minutes on ice) and rinsed twice with PBS. Staining was performed by incubating tissue sections for 1 hour at 37°C in a humidified chamber in the dark in 50 µl of TUNEL reaction mixture. DAPI counterstaining was used to improve image contrast. Negative controls were prepared by incubating fixed and permeabilized tissue sections in 50 µl of TUNEL label solution (without terminal transferase). Positive controls were prepared by incubating fixed and permeabilized tissue sections with DNase I Recombinant (2,500 U/ml in 50 mM Tris-HCl, pH 7.4, 1 mg/ml BSA, Roche Applied Science) for 10 minutes prior to labeling. Fluorescent images were acquired on a Nikon E600 imaging microscope.

#### Immunoblotting

Following each mechanical treatment, the specimens were pulverized using a mortar and pestle in liquid nitrogen, homogenized in ice-cold RIPA buffer (Santa-Cruz) and centrifuged at 7,000 g to pellet extracellular matrix debris for 8 minutes at 4°C. The supernatant was assayed for protein concentration using a bicinchoninic acid (BCA) protein assay (Pierce, Rockford, IL). Equal amounts of tissue lysates were resolved by reducing SDS-PAGE. After transfer to a polyvinylidene difluoride (PVDF) membrane (EMD Millipore) using a mini trans-blot cell (Bio-Rad), the blots were blocked with 5% non-fat drymilk and probed with a primary antibody against BMP-4 (1∶500, Abcam), TGF-β1 (1∶200, Santa Cruz), α-SMA (1∶200, Dako), Runx2 (1∶1500, Santa Cruz) or osteocalcin (1∶500, EMD Millipore). Depending on the primary antibody, appropriate anti-rabbit, anti-goat or anti-mouse HRP secondary antibody (1∶2000, Santa Cruz) was then used. The membranes were finally incubated in horseradish peroxidase-conjugated streptavidin. Immunopositive bands were then detected using a luminol-based chemiluminescence reagent (Pierce) against standard radiography film in a darkroom. The films were then analyzed by densitometry using the ImageJ program (NIH, Bethesda, MD).

#### Gelatin Zymography

Gelatin zymography was performed to quantify the activity of the proteolytic enzymes MMP-2 and MMP-9. Equal amounts of tissue lysates assayed by BCA were resolved by sample buffer (Bio-Rad) and loaded in the gel (Bio-Rad). After running, the gels were washed in 100 ml renaturation buffer (Bio-Rad) and developing buffer (Bio-Rad) overnight. The gels were stained by adding stain solution (Sigma-Aldrich), de-stained in deionized water, scanned and saved as a digital image. The images were then analyzed by densitometry using ImageJ.

#### Analysis of H&E Images

Routine H&E staining was used to assess tissue structure. Apart from a qualitative assessment, mean tissue thickness was determined from H&E micrographs by using the ImageJ software.

#### Semi-quantification of Immunostained Images

The semi-quantitative assessments of MMP-2, MMP-9, TIMP-2, cathepsin L and cathepsin S expressions were carried out using ImageJ. Briefly, the intensities of positively stained regions were estimated and normalized by the number of cells visible in the images to yield a quantity consistent to an expression of inflammatory marker per cell. The number of cells was estimated by splitting the microscope image captured using the triple filter of the mercury lamp into its red, green and blue channels, thresholding the blue channel image, and using the “analyze particles” function of ImageJ. The total expression of a given marker was assessed on microscope images captured by the camera using the green filter. Each image was split into its red, green and blue channels, and Imagej was used to compute the integral of the green channel histogram. Finally, the cellular expression was calculated in each image as the ratio of the total expression of marker to the number of cells. ImageJ was also used to quantify the results of the TUNEL assay. Cells with apoptotic fragments were detected using the green fluorescence of the microscope. The DAPI filter was used to image cell nuclei present on the micrograph. The apoptosis level was estimated as the ratio of the number of immunopositive cells to the total number of cells. Distinction was not made between endothelial and interstitial cells during the cell counting.

#### Densitometric Assessment of Immunoblots and Zymograms

The dedicated macro of ImageJ was used to plot the histogram of individual lanes/bands in blots/gels. These histograms were then integrated to obtain the mean intensity of each immunopositive band. For the blots, these intensities for a particular protein were then normalized by the intensity values for β-actin (Santa Cruz), which was used as a housekeeping protein.

### Statistical Analyses

All quantitative data were expressed as mean ± standard error. The sample size for each experimental group was n = 3. For each experimental condition, the quantitative and semi-quantitative results were averaged over the three samples to provide a mean cellular expression. All results were then normalized with respect to the values measured in fresh tissue (group 1), except for the analysis of TIMP/MMP ratio and for cellular apoptosis, which was expressed as the percentage of apoptotic cells relative to the total number of cells present in a sample. The data were first analyzed using ANOVA to determine if there was significant contribution by a particular mechanical treatment on the measured parameters, followed by the Bonferroni post-hoc test. A p-value of less than 0.05 was used as a measure of statistical significance. All statistical analyses were performed using SPSS (IBM, Armonk, NY).

## Results

### Leaflet Structure and Cell Viability Are Not Affected by TAV and BAV WSS

**Figure 4:**
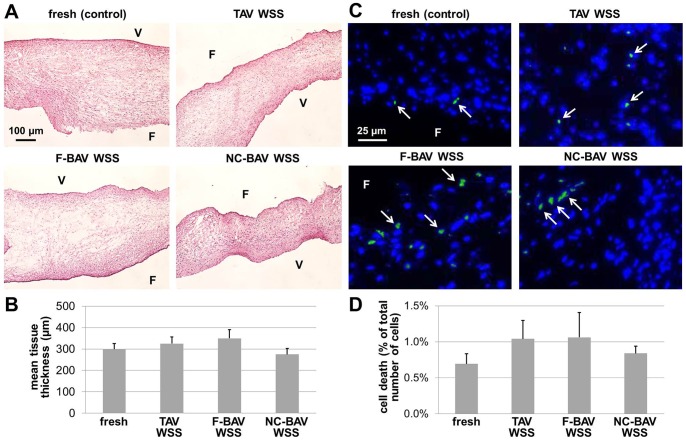
Effects of TAV and BAV WSS on tissue structure and cell viability. H&E stain (A), mean tissue thickness measurements (B), TUNEL assay (C) and quantitative TUNEL results (D) in porcine aortic valve leaflets subjected to TAV and BAV WSS (F: fibrosa; V: ventricularis, green: apoptotic cells, blue: cell nuclei).

Following tissue exposure to TAV and BAV WSS (groups 2, 3 and 4), the specimens were harvested and processed for standard H&E staining. Regardless of the mechanical treatment, no visible difference in tissue structure, cell density and leaflet thickness was detected between samples exposed to WSS and fresh controls (Figure 4A). Based on H&E staining alone, specimens conditioned under TAV and BAV WSS exhibited the same three-layered leaflet structure as that observed in fresh tissue. The quantification of the leaflet thickness with ImageJ in tissue from groups 2, 3 and 4 yielded average values of 328±29, 352±40, and 282±26 µm, respectively, which were not statistically different (p>0.06) from the thickness measured in fresh controls (302±25 µm) (Figure 4B). Lastly, the maintenance of cellular viability was verified by TUNEL assay. In all groups, apoptotic cell fragments were detected mainly in the sub-endothelial layer of the fibrosa (Figure 4C). Specimens exposed to TAV, F-BAV and NC-BAV WSS exhibited percentages of apoptotic cells (1.0±0.4%, 1.1±0.6% and 0.8±0.2%, respectively) statistically similar (p>0.90) to that measured in fresh tissue (0.7±0.2%) (Figure 4D). These results suggest that leaflet structure and cell viability are not affected by TAV and BAV WSS.

### BAV leaflet WSS Abnormalities Promote Fibrosa Endothelial Activation

**Figure 5:**
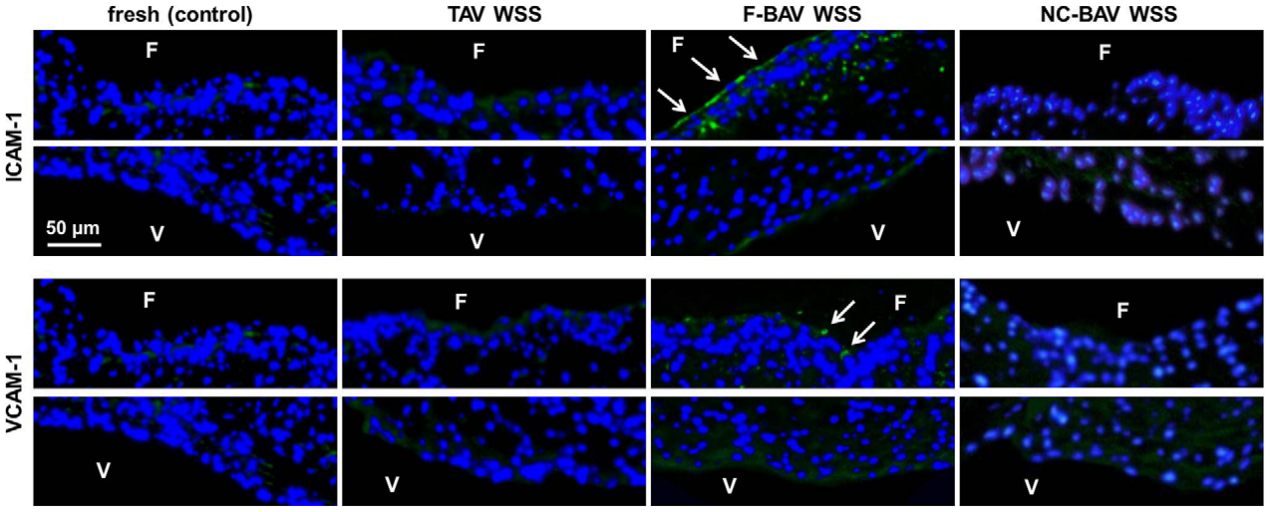
Effects of TAV and BAV WSS on endothelial activation. ICAM-1 and VCAM-1 immunostaining in porcine aortic valve leaflets subjected to TAV and BAV WSS (F: fibrosa; V: ventricularis; green: positively stained cells; blue: cell nuclei).

Immunostaining was performed to characterize the effects of TAV and BAV WSS on valvular endothelial activation in terms of ICAM-1 and VCAM-1 expression (Figure 5). Positive staining for ICAM-1 was only detected on the endothelial lining of the fibrosa in tissue subjected to F-BAV WSS conditions. Similarly, specimens exposed to F-BAV WSS exhibited a side-specific expression of VCAM-1, preferentially on the fibrosa. All other treatments did not result in any detectable change in cell-adhesion molecule expression relative to the fresh controls.

### Fused BAV Leaflet WSS Abnormalities Stimulate Paracrine Signaling via BMP-4- and TGF-β1-Dependent Pathways

**Figure 6:**
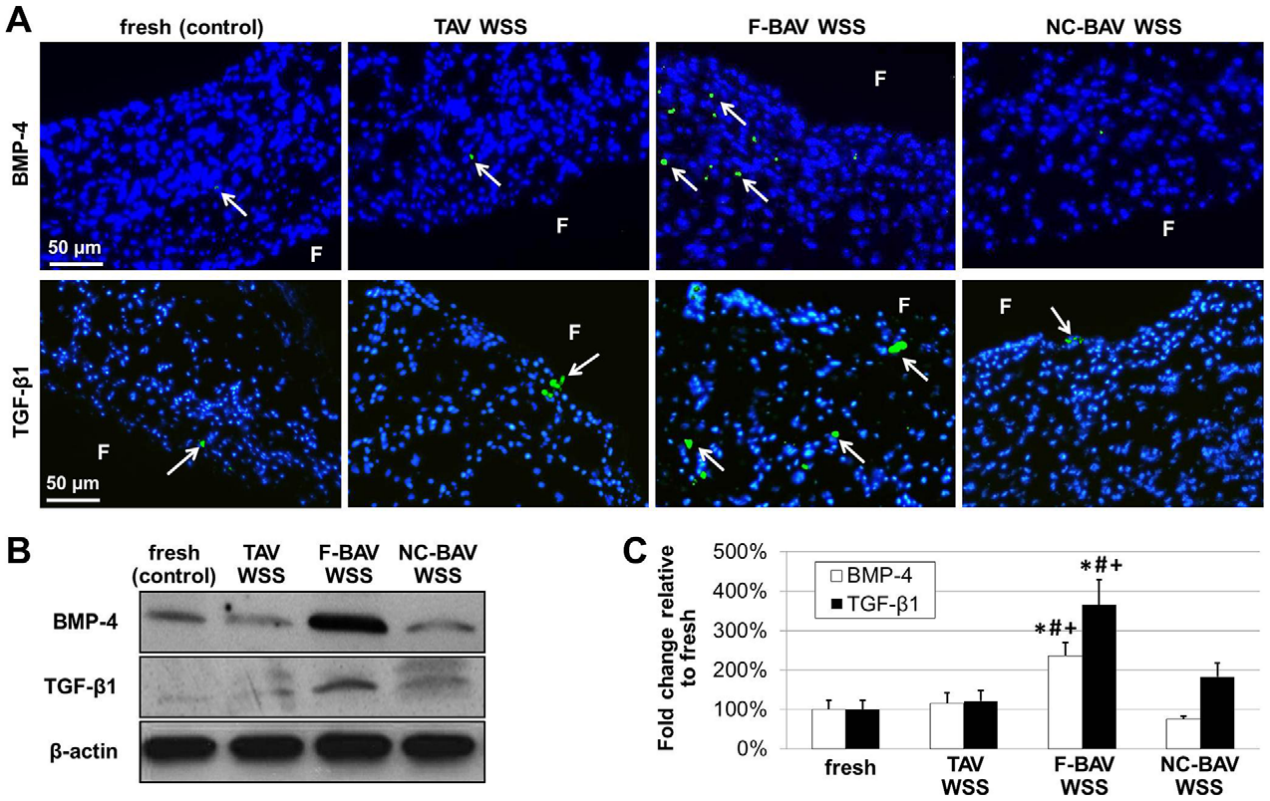
Effects of TAV and BAV WSS on paracrine signaling. BMP-4 and TGF-β1 immunostaining (A), immunoblotting (B) and densitometric results (C) in porcine aortic valve leaflets subjected to TAV and BAV WSS (F: fibrosa; green: positively stained cells; blue: cell nuclei; *: p<0.05 vs. fresh control; #: p<0.05 vs. TAV WSS; +: p<0.05 vs. NC-BAV WSS).

BMP-4 and TGF-β1 immunostaining was performed to investigate the potential of BAV WSS abnormalities to stimulate paracrine signaling pathways characteristic of the early stage of CAVD. While BMP-4- and TGF-β1-positive staining was detected near the endothelium in tissue exposed to TAV and NC-BAV WSS, expression levels were not qualitatively different from the baseline levels measured in fresh tissue (Figure 6A). In contrast, exposure of leaflet tissue to F-BAV WSS resulted in widespread BMP-4 and TGF-β1 expressions localized to the sub-endothelial layer of the fibrosa. Those results are supported by the Western blot results (Figure 6B and 6C) that suggest significant (p<0.05) increases in BMP-4 and TGF-β1 expression in tissue exposed to F-BAV WSS with respect to the fresh controls (2.4-fold and 3.7-fold increase, respectively). In contrast, BMP-4 and TGF-β1 levels measured in response to tissue conditioning under TAV and NC-BAV WSS were not statistically different from those in fresh tissue. Those results demonstrate that the specific WSS environment experienced by the F-BAV leaflet is able to stimulate paracrine signaling via BMP-4- and TGF-β1-dependent pathways.

### α-SMA and Runx2 Expressions Are Not Affected by TAV and BAV WSS

**Figure 7:**
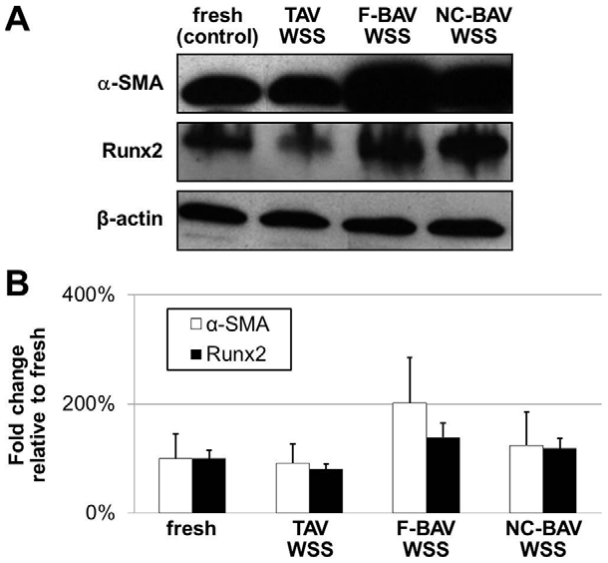
Effects of TAV and BAV WSS on α-SMA and Runx2 expressions. α-SMA and Runx2 immunoblotting (A) and densitometric results (B) in porcine aortic valve leaflets subjected to TAV and BAV WSS.

The effects of BAV WSS abnormalities on VIC differentiation mechanisms were investigated by measuring α-SMA and Runx2 expressions following tissue exposure to TAV and BAV WSS. α-SMA is a non-specific indicator of the myofibroblast phenotype, while Runx2 is a key transcription factor associated with osteoblast differentiation and is also a downstream transcription factor for BMP-4. While the trends emerging from the immunoblotting data suggested increased α-SMA expression in response to F-BAV and NC-BAV WSS (2-fold, 1.2-fold increase, respectively) relative to the level measured in fresh controls, the absence of statistical significance suggested that TAV and BAV WSS essentially maintained α-SMA expression level (Figure 7). Similarly, while Runx2 expression exhibited a similar trend (1.3-fold and 1.2-fold increase in response to F-BAV and NC-BAV WSS, respectively), the upregulation was not statistically significant. While gene expression studies are required to confirm this observation, those results suggest that BAV WSS abnormalities did not trigger molecular mechanisms associated with VIC myofibroblastic and osteoblastic differentiation over the short duration of our experiments.

### BAV WSS Abnormalities Promote ECM Remodeling via MMP- and Cathepsin-Dependent Pathways

**Figure 8:**
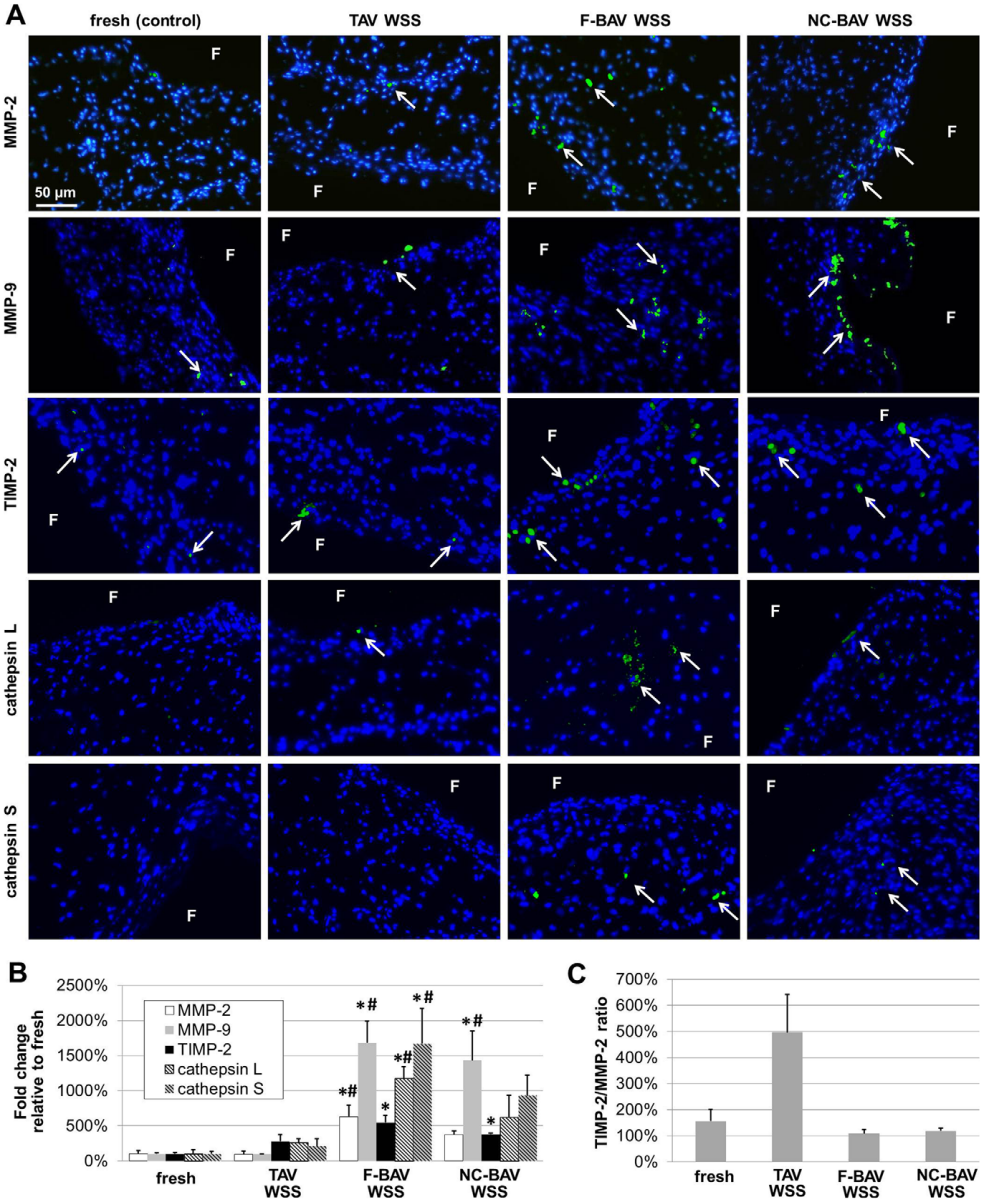
Effects of TAV and BAV WSS on valvular remodeling. MMP-2, MMP-9, TIMP-2, cathepsin L and cathepsin S immunostaining (A), semi-quantitative results (B) and TIMP-2/MMP-2 ratio (C) in porcine aortic valve leaflets subjected to TAV and BAV WSS (F: fibrosa; green: positively stained cells; blue: cell nuclei; *: p<0.05 vs. fresh control; #: p<0.05 vs. TAV WSS).

**Figure 9:**
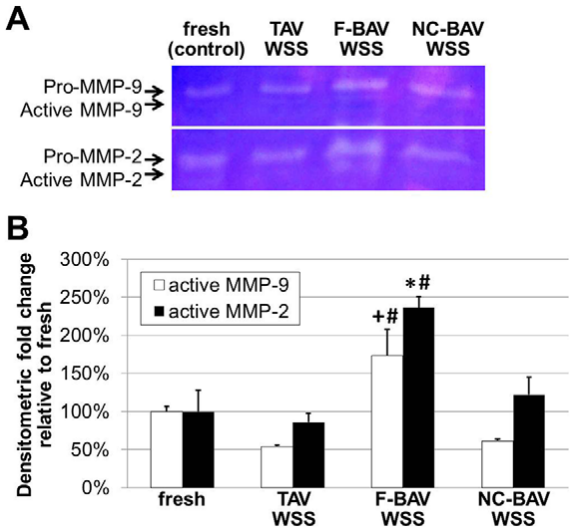
Effects of TAV and BAV WSS on gelatinase activity. MMP-2 and MMP-9 gelatin zymography (A) and active MMP-2 and MMP-9 semi-quantitative results (B) in porcine aortic valve leaflets subjected to TAV and BAV WSS (*: p<0.05 vs. fresh control; #: p<0.05 vs. TAV WSS; +: p<0.05 vs. NC-BAV WSS).

The downstream effects of BAV hemodynamic abnormalities on valvular remodeling were investigated by comparing the secretion of the proteolytic enzymes MMP-2, MMP-9, their tissue inhibitor TIMP-2, and the elastolytic proteases cathepsin L and cathepsin S in the different tissue groups. Immunostaining results (Figure 8A) suggested increased enzymatic expressions in tissue exposed to BAV WSS as compared to those measured in tissue exposed to TAV WSS and in fresh controls. In addition, those molecules were primarily detected in the sub-endothelial layer of the fibrosa. Tissue exposed to TAV and BAV WSS consistently exhibited more TIMP-2-positive cells than fresh tissue and, when detected, TIMP-2 expression was localized in the leaflet sub-endothelial layers. The semi-quantitative analysis of the immunostaining results supported those observations (Figure 8B). F-BAV WSS abnormalities resulted in a significant increase in MMP-2, MMP-9, cathepsin L, cathepsin S and TIMP-2 expression relative to fresh controls (6.3-fold, 16.8-fold, 11.7-fold, 16.7-fold and 5.5-fold respectively; p<0.05). While the exposure of leaflet tissue to NC-BAV WSS also resulted in the increased expression of each biomarker relative to the fresh controls, only MMP-9 and TIMP-2 were significantly upregulated (14.2-fold and 3.8-fold increase, respectively; p<0.05). In contrast, TAV WSS maintained MMP and cathepsin expressions at the same levels as those in fresh tissue. The impact of the different WSS environments on the remodeling state of the tissue was also investigated by examining TIMP-2-to-MMP-2 ratio (Figure 8C). Interestingly, while no statistical difference was found between the groups, the overall trend indicated higher ECM remodeling potential (i.e., smaller TIMP/MMP ratio) in response to F-BAV WSS (TIMP/MMP = 109%) and NC-BAV WSS (TIMP/MMP = 118%) than in response to TAV WSS (TIMP/MMP = 497%) or in fresh tissue (TIMP/MMP = 156%). Gelatin zymography was carried out to quantify the active forms of MMP-2 and MMP-9 in the different tissue groups (Figure 9). The densitometric analysis of the zymograms revealed a significant increase in proteolytic enzyme activity in tissue exposed to F-BAV WSS as compared to fresh tissue (1.7-fold and 2.4-fold increase in MMP-2 and MMP-9 activity, respectively; p<0.05). Those results suggest that BAV WSS abnormalities tend to break valvular homeostasis by upregulating catabolic enzyme expression and activity, and creating an imbalance between catabolic enzymes and their tissue inhibitors.

### Fused BAV Leaflet WSS Abnormalities Promote Osteocalcin Expression

**Figure 10:**
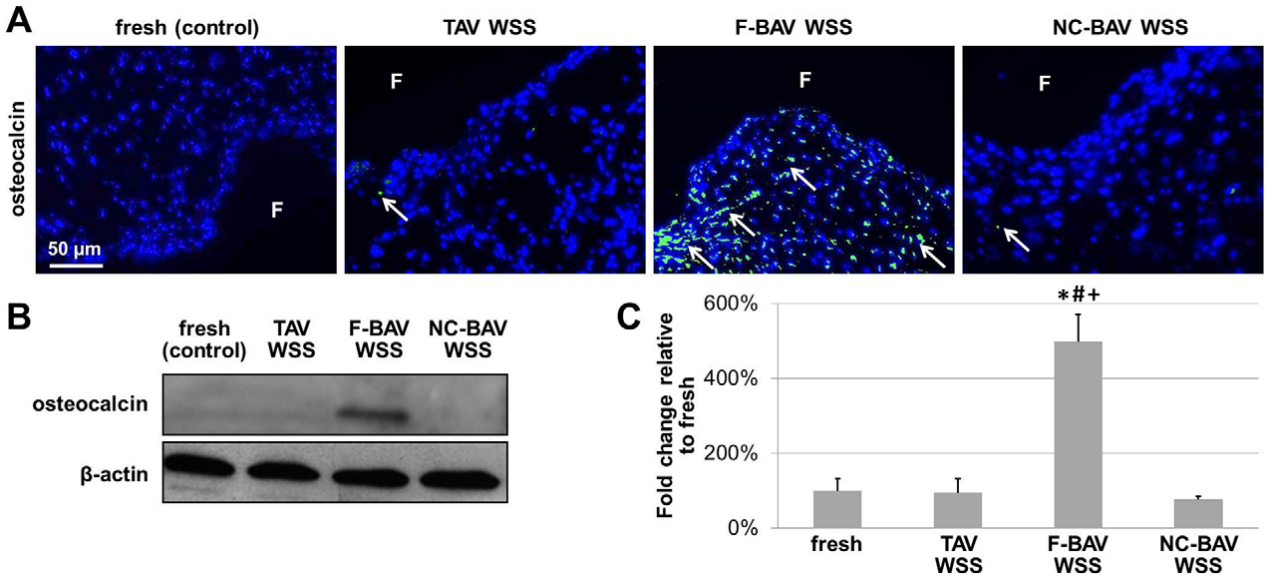
Effects of TAV and BAV WSS on valvular osteogenesis. Osteocalcin immunostaining (A), immunoblotting (B) and densitometric results (C) in porcine aortic valve leaflets subjected to TAV and BAV WSS (F: fibrosa; green: positively stained cells; blue: cell nuclei; *: p<0.05 vs. fresh control; #: p<0.05 vs. TAV WSS; +: p<0.05 vs. NC-BAV WSS).

The investigation of bone matrix synthesis following VIC osteoblastic differentiation was performed via the detection of osteocalcin, a glycosylated phosphoprotein important in skeletal bone mineralization. Immunostaining results (Figure 10A) indicate abundant osteocalcin expression preferentially localized to the fibrosa and spongiosa of tissue exposed to F-BAV WSS. In contrast, fresh tissue and tissue exposed to TAV and NC-BAV WSS expressed little amount of osteocalcin. Consistent with those observations, the Western blot results suggest a 5-fold increase (p<0.05) in osteocalcin expression between tissue exposed to F-BAV WSS and fresh controls (Figure 10B). In contrast, similar osteocalcin levels were detected in fresh tissue, tissue exposed to TAV WSS and tissue exposed to NC-BAV WSS. These data suggest the higher osteogenic susceptibility of the F-BAV leaflet to secrete bone-like matrix in response to its abnormal WSS environment.

## Discussion

Despite the compelling evidence for the involvement of hemodynamic stress abnormalities in valvular disease, the elucidation of the role played by the native BAV hemodynamics in CAVD development has been hampered by its complexity and the challenge to replicate it in the laboratory setting. Using an *ex vivo* approach enabling the exposure of aortic valve leaflets to *in vivo*-like TAV and BAV WSS, we demonstrated the potential of BAV hemodynamic stresses to trigger biological events marking the early stage of CAVD, namely endothelial activation, pro-inflammatory paracrine signaling, ECM degradation and possibly osteogenesis. The differential tissue responses following exposure to TAV, F-BAV and NC-BAV WSS confirm the sensitivity of aortic valve leaflets to their surrounding fluid shear stress environment and indicate the pathogenic potential of the native BAV hemodynamic environment as well as the particular vulnerability of the F-BAV leaflet to calcification.

The main contribution of this study is the demonstration of the key role played by BAV hemodynamic stress abnormalities in the initiation and progression of CAVD. The use of normal aortic valve leaflet tissue in the experiments permitted to eliminate the possible involvement of any intrinsic genetically programmed biology in the biological responses. Therefore, the only factor that could possibly produce the differential biological outcomes described in this study is the mechanical treatment to which each tissue group was subjected. Another important contribution is the apparent dependence of the severity of the pathological response on the imposed degree of WSS abnormality. While leaflets conditioned under NC-BAV WSS essentially exhibited the same biological signature as fresh tissue or leaflets conditioned under TAV WSS, leaflets exposed to F-BAV WSS demonstrated upregulation in all CAVD biomarkers except those associated with VIC differentiation mechanisms (i.e., α-SMA and Runx2). Interestingly, our previous characterization of TAV and BAV hemodynamics had revealed the existence of drastic differences in WSS magnitude, which attain a maximum in the base of the fibrosa (600% and 240% increase in WSS magnitude on the F-BAV and NC-BAV leaflet, respectively, relative to the TAV leaflet) and become increasingly significant during valvular sclerosis [55]. Clinical studies have also shown that the F-BAV leaflet is the most common site of calcific lesion formation in type-I BAV [56]. This parallel provides compelling support to the hemodynamic theory of CAVD in the BAV and suggests that BAV hemodynamics may contribute to the rapid and severe development of calcific lesions by imposing abnormally elevated WSS in the base of the F-BAV leaflet.

Of particular interest is the side-specific expression of cell-adhesion molecules on the fibrosa following leaflet exposure to BAV WSS abnormalities. This result is consistent with the previously established existence of pro- and anti-inflammatory VEC phenotypes on the leaflet fibrosa and ventricularis, respectively, which contribute to the specific vulnerability of the leaflet fibrosa to calcification [48]. In addition to this side-specificity, our results indicate that WSS-induced endothelial activation is leaflet-specific in the BAV and occurs primarily on the fused leaflet. This observation can be explained by the leaflet-specific WSS environment present in the BAV, which is characterized by highly elevated and mildly elevated WSS magnitudes on the fibrosa of the fused and non-coronary leaflets, respectively, relative to TAV leaflets (6-fold and 2.4-fold increase, respectively) [55] and the previously established ability of supra-physiologic WSS levels to trigger endothelial activation in a WSS magnitude-dependent manner [47].

A striking observation is the magnitude of the pathological response following the acute exposure of normal valve leaflets to BAV hemodynamic abnormalities. While this response does not seem consistent with the relatively slow progression of CAVD, which occurs over years *in vivo* [66], [67], this accelerated pathogenesis may be explained by the particular *ex vivo* environment produced by the shear stress device, which purposely eliminated all mechanical cues normally experienced *in vivo* but WSS. *In vivo*, the difference of pressure across the valve generates a compression force normal to the leaflet fibrosa, which varies cyclically during the cardiac cycle and attains a peak during diastole [68]. The deformation of the leaflets in response to this cyclic pressure in turn generates an axial stretch, which affects the length of the leaflet [68]. While pressure and stretch are critical to proper valvular function and coaptation, they also play critical roles in valvular homeostasis and disease by regulating ECM biosynthesis and degradation, cellular proliferation and differentiation, and inflammatory processes. Collagen, sulfated glycosaminoglycan (sGAG) and DNA syntheses have been shown to be modulated in pressure and stretch magnitude- and frequency-dependent manners [58], [59]. While pulsatile pressure tends to downregulate VIC differentiation into myofibroblasts [59], [69], cyclic stretch was shown to upregulate this contractile phenotype [58]. In addition, the expression and activity of proteolytic enzymes such as cathepsins and MMPs, which play important roles in CAVD pathogenesis, were shown to be mechanosensitive to stretch [34]. Lastly, elevated pressure and stretch levels have been shown to modulate valvular inflammation via BMP-4- and TGF-β1-dependent pathways [57], [70]–[72]. Those studies suggest that valvular homeostasis is maintained, at least partially, by synergies between the mechanical signals received by the leaflets *in vivo*. Therefore, the absence of the compensatory mechanisms regulated by stretch and pressure may have compromised valvular homeostasis and amplified the pathological response observed in our experiments.

In addition, our results provide further insights into the role played by VECs and VICs in WSS mechanotransduction. As compared to stretch and pressure, which propagate throughout the leaflet and stimulate both VECs and VICs, WSS is an interfacial stress sensed primarily by VECs. Interestingly, while BAV WSS abnormalities upregulated cell-adhesion molecule expression in VECs lining the leaflet fibrosa, all other downstream effects (i.e., cytokine expression, ECM remodeling, osteogenesis) are VIC-specific processes. This observation suggests that VECs are able not only to sense WSS abnormalities but also to transduce those signals deeper in the tissue by altering VIC function. This is consistent with recent studies that have evidenced the existence of an intricate communication network between the two cell types, as illustrated by the difference in collagen and sGAG synthesis between intact and endothelium-denuded leaflet tissue subjected to fluid shear stress [45], the change in interstitial cell phenotype and matrix synthesis between intact and endothelium-deprived VIC-VEC co-culture models exposed to steady WSS [73], and the modulation of valvular ECM remodeling by WSS magnitude [74]. Although the mode of communication between the two cell types remains largely unknown, paracrine expression has been suggested as a potential mechanism. *Ex vivo* studies have shown the upregulation of pro-inflammatory cytokines in the leaflet interstitium following endothelial exposure to abnormal WSS magnitude or pulsatility [46], [47]. Therefore, the increased BMP-4 and TGF-β1 expressions detected in response to BAV WSS abnormalities provides further evidence of the involvement of paracrine signaling in VEC-VIC communication and its role in the transduction of WSS abnormalities into a pathological response.

Another interesting point is the apparent contrast between the low sensitivity of α-SMA and Runx2, as suggested by the absence of statistical significance between the different tissue groups, and the global increase in pro-calcific potential observed in response to BAV WSS. One explanation for this apparent inconsistency may be related to the short duration of our experiments. While α-SMA and Runx2 are not specific markers of VIC myofibroblastic and osteoblastic differentiations, the maintenance of their expression levels may indicate the non-active state of those processes. Since VIC activation is an event occurring relatively late in the calcification pathway, the absence of significant changes in the expression of these molecules in the acute response described in this study was expected. Complementary studies focusing on gene and ED-A fibronectin expression (i.e., cellular fibronectin expressed by fibroblasts at very early stages of differentiation into myofibroblasts) and calcium assay are needed to examine this hypothesis.

Lastly, although experimental and analytical techniques have been used to estimate TAV and BAV leaflet WSS [75]–[78], our choice to consider predicted rather than measured WSS values was based on the requirement to expose the circular leaflet specimens to their *local* WSS. As compared to the experimental techniques, which provide WSS estimates at a point or along a line, the use of a computational model [55] permitted to generate WSS data over the *exact* leaflet region from which the circular tissue samples were extracted. In addition, while the experimental approach should always be considered the gold standard for flow characterization, it may not be the most suitable for the calculation of spatially dependent quantities such as WSS, especially on moving valve leaflets. In fact, experimental uncertainties in the estimation of the distance between the location of the velocity measurement and the moving wall and in the measurement of near-wall flow velocities may compromise the calculation of the spatial velocity gradient near the leaflet surface and, in turn, the estimation of the WSS. Those considerations justify the need for a better characterization of valvular WSS.

In summary, the results presented in this study clearly demonstrate the pro-calcific potential of the hemodynamic abnormalities produced by the BAV anatomy. Our data indicate that the native WSS abnormalities present on the F-BAV leaflet promote endothelial activation on the fibrosa, pro-inflammatory paracrine signaling and catabolic enzyme secretion, and bone matrix synthesis. Although the results seem to point to the existence of correlations between the local degree of WSS abnormality on BAV leaflets and leaflet vulnerability to CAVD, further investigations are required to demonstrate causality. The results of this study also suggest that calcific BAV disease may not solely result from a particular genetic predisposition to calcification but from complex synergies between the intrinsic genetically programmed biology of BAV tissue and the hemodynamic abnormalities experienced by BAV leaflets.

### Limitations

The experimental duration considered in this study was limited to 48 hours. While leaflet calcification in both the TAV and the BAV typically occurs over a much longer timescale than that considered in this study, the present results suggest acute differences in the biological responses of leaflet tissue exposed to TAV and BAV WSS. Those acute effects are necessary for the characterization of the contribution of BAV hemodynamic abnormalities to valvular calcification since they may shape the longer-term mechanisms involved in the progression of valvular disease. Nevertheless, the ability to extend the experimental duration would provide a more complete map of the processes involved in the formation of BAV calcific lesions.

The *ex vivo* approach adopted in this study enabled the characterization of the valvular mechano-sensitive response to TAV and BAV WSS while preserving the native architecture of the leaflets (i.e., VICs and VECs embedded in their extracellular matrix). Although this approach provides insights into the global tissue response resulting from the native cell-cell and cell-ECM interactions, it is not capable of providing a mechanistic description of those processes or isolating the response of each cell type. Gene and cell studies are needed to better characterize the signaling pathways triggered by WSS abnormalities.

Lastly, it is important to recognize that although the biological results described in this paper might translate to adult human valves, one should account for the intricate differences between porcine and human valve tissues and their respective biology. In this context, while this study suggests a link between BAV hemodynamic abnormalities and early calcification mechanisms, the generalization to human valvular disease pathogenesis requires complementary experiments with human tissue.
